# Stiffness Estimates for Composites with Elliptic Cylindrical Voids

**DOI:** 10.3390/ma13061354

**Published:** 2020-03-17

**Authors:** Fabian Becker, Christian Hopmann

**Affiliations:** Institute for Plastics Processing, Fakultät für Maschinenwesen, RWTH Aachen University, Seffenter Weg 201, 52074 Aachen, Germany; sekretariat@ikv.rwth-aachen.de

**Keywords:** fiber-reinforced plastics, voids, Porosity, composites, Mori–Tanaka, Eshelby tensor, transversal isotropic

## Abstract

A two-step homogenization procedure is presented to investigate the stiffness of a unidirectional continuous fiber-reinforced composite material containing voids of different shapes and volume contents. Since the Mori–Tanaka scheme is limited to moderate volume contents of the inhomogeneity phase, fiber and matrix are homogenized with semi-empirical relations with use of the adjusted fiber volume content in a first step. In the second step, the Mori–Tanaka scheme is applied to obtain the homogenized stiffness tensor of a transversely isotropic material containing voids aligned with the fiber direction. The voids are modelled with infinite length, but an elliptic base characterized by the aspect ratio. The tensor components of the Eshelby tensor for this case are presented in closed form for a transversely isotropic material depending on the aspect ratio and matrix material properties. The scheme is solved directly for easy implementation and the use of fast calculations of the effective engineering constants of a composite material containing voids. Experimental results from literature for different void contents and shapes are compared to the predicted moduli with cylindrical voids. From the results it is further concluded that the aspect ratio of the void and the manufacturing process of the composite should be considered.

## 1. Introduction

Voids in fiber-reinforced composites are common manufacturing defects and influence the resulting stiffness and strength of the material. In recent decades, numerous researchers investigated on the influence of voids on the resulting properties of composite materials. Early experimental investigations by Lenoe focused on the effects of voids on the mechanical properties for unidirectional and quasi-isotropic laminates for carbon fiber epoxy composites [1]. Void contents were estimated between 1% and 5%. Longitudinal, transverse tension and shear tests were conducted. However, no correlation function for void content and resulting properties was established. Experimental investigations on the influence of voids on the flexural performance were performed for unidirectional glass fiber-reinforced polypropylene by Hagstrand et al. [2]. They found a linear relationship between the void content and the decrease in the effective modulus in fiber direction. (1.5% loss in bending stiffness for 1% higher void content).

Varna et al. investigated a unidirectional glass fiber vinylester pre-preg material containing voids [3]. The resulting fiber volume content was 66%, with the void content range from 0% to 5%.

Gürdal et al. investigated quasi-isotropic laminate lay-ups and the influence of voids on the mechanical properties [4]. They related the strength to the void content. Liu et al. manufactured cross ply laminates and evaluated the flexural modulus and the tensile modulus [5]. They used carbon fiber epoxy specimens with a fiber volume content of 60%±2%. The void volume content was varied between 0.5% and 3.5%. They reported that the tensile modulus is insensitive to voids. However, a linear relationship with a low gradient between the tensile modulus and the void content could be obtained.

Other experimental investigations including the consideration of the shape of a void are found in the review article of Mehdikani et al. [6]. The voids are characterized as micro-, meso and macro-voids with different shapes. The relevant meso and macro-voids often show a pancake-like appearance, with the main axis of the voids aligned to the fiber direction. The dimension in fiber direction is significantly larger than in the lamina plane.

The aim of the research presented is to propose a method to calculate the resulting engineering constants of a composite material including elliptic cylindrical voids. The method is based on a two-step homogenization procedure.

Two-step homogenization procedures have been employed by a lot of authors in the past. Peng et al. [7] used a self-consistent scheme in the first step and the Mori–Tanaka estimate in the second step to calculate the resulting properties of a particulate composite with different constituents. Kushch et al. developed a solution for multiple interacting inclusions based on potential theory and homogenization with the Maxwell scheme [8]. Barboura et al. used a gradual two-step homogenization scheme [9]. Cohen et al. estimated the transverse modulus of a three-phase composite with void and a rectangular filler embedded in matrix material. They proposed a very simple two-step homogenization scheme [10]. The double inclusion method for the effective elastic properties of elastic particles embedded in another elastic phase has been developed by Hori et al. [11].

The method presented within this article employs simple mixing rules in the first step to calculate the engineering constants of a composite - with respect to the processing route. The void content and the effect of the voids’ simplified geometry is subsequently homogenized with the composite phase via the Mori–Tanaka scheme.

For the use in the first step, various approaches and homogenization procedures have been proposed in the past. For transverse moduli of a composite, the simple rules of mixtures assuming same stress or same strain in the constituents give the Reuss-Voigt bounds [12]. Narrower bounds, without considering the shape of the composite constituents, give the Hashin–Shriktman bounds [13]. Semi-empirical models like Halpin-Tsai model can be connected with Hashin–Shriktman bounds to give reasonable estimates of the fit parameters to calculate engineering moduli [14].

McLaughlin and Norris applied the differential homogenization scheme to transversely isotropic materials [15,16]. Equations for the calculation of the in-plane Poisson ratio and for the in-plane shear modulus for unidirectional layers were established by Craig et al. [17]. The transverse through thickness Poisson ratio can be calculated as published by Christensen [18] or Philippidis et al. based on the stiffness tensor [19].

Tandon and Weng used the Mori-Tanka Scheme for estimation of the engineering constants with aligned spheroidal inclusions for different aspect ratios being defined via the length of the inclusion. They apply their solution to calculate the resulting properties of a composite consisting of fibers in an isotropic matrix based on the work of Mura [20,21]. Benveniste et al. also applied the Mori–Tanaka scheme to calculate the resulting composite properties [22]. Ju and Chen derived effective composite properties based on a noninteracting solution of the eigenstrains [23]. Other dilute or self-consistent homogenization schemes based on the Eshelby solution are found in the literature review of Tucker et al. [24].

Fit equations from finite element calculations for calculation of the transverse Young’s modulus and shear modulus are presented by Bleier [25].

Huang et al. applied the Mori–Tanaka Scheme to compare the results of the finite element method (FEM) to experimental data of composite specimens with voids of different aspect ratios [26]. The Eshelby tensor was integrated from Green’s function numerically. However, they did not present the Poisson numbers and did not differentiate between different processing routes. Farouk et al. derived the change in modulus by voids via application of a multi-phase equivalent inclusion homogenization [27]. Chao et al. used the same method to obtain the elastic constants for porous aluminum composites [28].

Chiang proposed an asymptotic closed form analytical expression for the general ellipsoidal case of an inclusion with three different semi-axes of the inclusion [29]. The closed form has been presented in the same paper of Chiang for an infinite cylinder with circular basis. The results contain the solution of Whiters, who gave closed form expressions for the Eshelby tensor for a transversely isotropic matrix and a spheroidal inclusion [30]. The resulting properties of the Mori–Tanaka scheme are not reliable when it comes to higher fiber volume fractions, since the fiber distribution and the interaction of the fibers are not accounted for correctly. However, the void content found in composite materials does not exceed moderate values. The Mori–Tanaka scheme therefore is applicable for the integration of voids into a transversely isotropic matrix in a second step, with the properties of the matrix being the properties of a composite. With the recent closed form formulation of the Eshelby tensor by Chiang et al. [29], it is now possible to separate the two homogenization steps and to obtain easy-to-use equations for the calculation of the elastic constants of composites with voids.

## 2. Modelling

**Figure 1:**
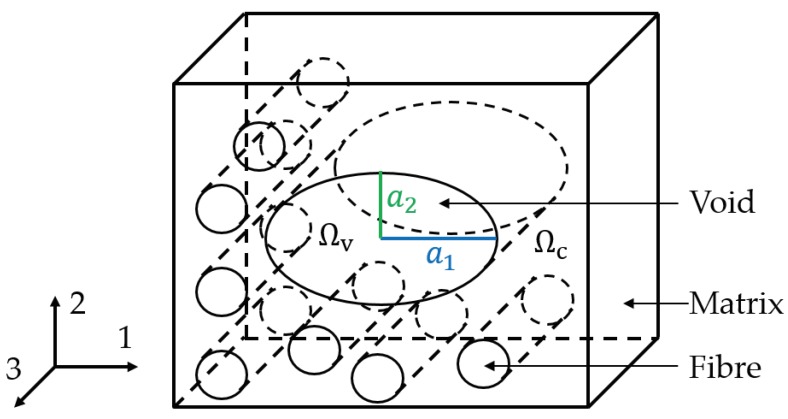
Definition of fiber and void orientations and their domains.

Composite materials consist of fibers in a matrix phase containing voids. The fiber direction is defined as “3” direction and the transverse directions are known as “1” direction (in the lamina plane) and “2” direction in the thickness direction. Voids show a much larger dimension in fiber direction compared to the other directions [4,31]. Therefore, the voids are modelled as cylinders with infinite “3” axis but different semi-axes in “1” and “2” directions. For the scope of the research presented, the void semi-axes are aligned to the “1” and “2” directions. The domain within the void is called “void domain ($\Omega_{v}$)” and the region outside the void is called “composite domain ($\Omega_{c}$)” (Figure 1). All properties regarding the composite domain or the void domain are indexed with “*c*” or “*v*”.

The aim is to investigate the influence of different void geometries and void contents on the properties of the fiber-reinforced composite. For the solution of the problem, the overall stiffness tensor of the composite with voids of different shapes and contents must be determined to calculate the resulting engineering properties.

### 2.1. Two-Step Homogenization Scheme

To overcome the limitations of the Mori–Tanaka scheme, a two-step homogenization approach is developed. The approach presented uses the solution of the Eshelby tensor in a transversely isotropic matrix and is different from a two-step homogenization where the Mori–Tanaka method is applied in two subsequent steps or a multi-inclusion method in isotropic matrix as described by Nemat et al. [32]. In the first step, fiber and matrix are homogenized with a semi-empirical relationship for the engineering constants of a composite calculated from the properties of fiber and matrix. Since the first homogenization step only focusses on the domain outside the void, special attention is drawn to the fiber volume content of the composite domain ($\varphi_{f}^{*}$). The “adjusted” fiber volume content in the composite domain can reach higher values than the global fiber volume content ($\varphi_{f}$), depending on the manufacturing process. An increasing fiber volume content in the composite domain is a reasonable assumption for all manufacturing processes of composites, where the amount of fibers per part is controlled as well as the part’s geometry. This applies to manufacturing processes like resin transfer molding (RTM) or pultrusion processes. In these processes, the voids are found as meso or macro-voids in the matrix phase. For manufacturing processes that allow for a change in cross section geometry of the specimens, like filament winding without thickness control or hand lay-ups, the global fiber volume content should be used instead, since the voids’ cross section adds to the total cross section of a specimen, without changing the fiber volume content of the composite domain. However, this is seen to be a lower bound here, since different pressure profiles during curing e.g., in autoclave processes may lead to a fiber volume content in between the two bounds depending on the void volume content.

(1) $$\begin{array}{c} \varphi_{f}^{*}\approx \left\{\begin{array}{cc} \frac{\varphi_{f}}{1-\varphi_{v}} & e.g.,\;\text{for pultrusion or RTM processes}\\ \varphi_{f} & e.g.,\;\text{for filament winding} \end{array}\right. \end{array}$$

With the adjusted fiber volume content, the resulting properties are calculated assuming transversely isotropic behavior of the composite domain. For the Young’s modulus in fiber direction the mixing rule is used, where index “f,ij” stands for fiber stiffness tensor components in “i,j” and “*m*” denotes the matrix properties:

(2) $$E_{33}^{c}=E_{f,33}\cdot \varphi_{f}^{*}+E_{m}\cdot \left(1-\varphi_{f}^{*}\right)$$

The remaining engineering constants are calculated within this article with the equations published and investigated by Bleier [25]. However, the method is not limited to the use of this specific rules. Other semi-empirical or self-consistent calculation schemes may be used (compare [17,19,24,32,33]).

(3) $$\begin{array}{ccc} E_{11}^{c} & = & E_{22}^{c}=\frac{E_{m}}{2}\cdot \frac{1+f_{\varepsilon }^{0.68}}{\left(1-\varphi_{f}^{*}\right)+\sqrt{\varphi_{f}^{*}}\cdot \left(1-\frac{E_{m}}{E_{f,11}}\right)} \end{array}$$

(4) $$\begin{array}{ccc} G_{13}^{c} & = & G_{23}^{c}=G_{m}\cdot \frac{0.67+0.33\cdot f_{\gamma }^{0.89}}{\left(1-\varphi_{f}^{*}\right)+\varphi_{f}^{*}\cdot \sqrt{\frac{G_{m}}{G_{f,32}}}} \end{array}$$

With the following factors:

(5) $$\begin{array}{ccc} f_{\varepsilon } & = & \frac{1}{1-\frac{2}{\sqrt{\pi }}\cdot \sqrt{\varphi_{f}^{*}}\cdot \left(1-\frac{E_{m}}{E_{f,11}}\right)} \end{array}$$

(6) $$\begin{array}{ccc} f_{\gamma } & = & \frac{1}{1-\frac{2}{\sqrt{\pi }}\cdot \sqrt{\varphi_{f}^{*}}\cdot \left(1-\frac{G_{m}}{G_{f,23}}\right)} \end{array}$$

Under the assumption of transverse isotropy in the composite domain, the modulus $G_{12}$ can be calculated as:

(7) $$G_{12}^{c}=\frac{E_{22}^{c}}{2\;(1+\nu_{12}^{c})}$$

With the Poisson number $\nu_{12}^{c}$ following Christensen’s calculation from transverse isotropy of the composite domain [18]:

(8) $$\nu_{12}^{c}=\nu_{31}^{c}\cdot \frac{1-\nu_{13}^{c}}{1-\nu_{31}^{c}}$$

The remaining Poisson numbers are calculated with the rule of mixtures and with the help of the symmetry of the stiffness tensor:

(9) $$\begin{array}{ccc} \nu_{31}^{c} & = & \nu_{f,31}\cdot \varphi_{f}^{*}+\nu_{m}\cdot \left(1-\varphi_{f}^{*}\right) \end{array}$$

(10) $$\begin{array}{ccc} \nu_{13}^{c} & = & \frac{E_{11}^{c}}{E_{33}^{c}}\cdot \nu_{31}^{c} \end{array}$$

Improvements to the quality of the calculated engineering constants in the first step are not the focus within this article and may be found elsewhere [17,19,34]. However, all calculation rules show a reasonable agreement compared to the finite element solution, except for differences of the Poisson number $\nu_{12}^{c}$ (see Section 4). For autoclave processes, when the assumption of the adjusted fiber volume content being equal to the global fiber volume content is reasonable, the first homogenization step may be omitted by measurements of the moduli and Poisson numbers. Since all engineering constants for the unidirectional continuous fiber composite domain are now defined, the fourth rank compliance tensor can be unfolded with the Mandel (Kelvin) notation, for a transversely isotropic material with “3” being along the six-fold symmetry [35]:

(11) $${C^{c}}^{-1}=\left[\begin{array}{cccccc} \frac{1}{E_{11}^{c}} & -\frac{\nu_{12}^{c}}{E_{11}^{c}} & -\frac{\nu_{13}^{c}}{E_{11}^{c}} & 0 & 0 & 0\\ -\frac{\nu_{12}^{c}}{E_{11}^{c}} & \frac{1}{E_{11}^{c}} & -\frac{\nu_{13}^{c}}{E_{11}^{c}} & 0 & 0 & 0\\ -\frac{\nu_{13}^{c}}{E_{11}^{c}} & -\frac{\nu_{13}^{c}}{E_{11}^{c}} & \frac{1}{E_{33}^{c}} & 0 & 0 & 0\\ 0 & 0 & 0 & \frac{1}{2\;G_{13}^{c}} & 0 & 0\\ 0 & 0 & 0 & 0 & \frac{1}{2\;G_{13}^{c}} & 0\\ 0 & 0 & 0 & 0 & 0 & \frac{1}{2\;G_{12}^{c}} \end{array}\right]$$

The elasticity tensor can be noted in Mandel (Kelvin) notation as:

(12) $$C^{c}=\left[\begin{array}{cccccc} C_{11}^{c} & C_{12}^{c} & C_{13}^{c} & 0 & 0 & 0\\ C_{12}^{c} & C_{11}^{c} & C_{23}^{c} & 0 & 0 & 0\\ C_{13}^{c} & C_{23}^{c} & C_{33}^{c} & 0 & 0 & 0\\ 0 & 0 & 0 & C_{44}^{c} & 0 & 0\\ 0 & 0 & 0 & 0 & C_{44}^{c} & 0\\ 0 & 0 & 0 & 0 & 0 & C_{66}^{c} \end{array}\right]$$

In a second step, the Mori–Tanaka solution is used, where the (already homogenized) composite domain and the void domain are homogenized. The Mori–Tanaka scheme results in the following equation [36], with bold symbols denoting the fourth rank tensors:

(13) $$C^{\mathrm{mt}}=\left(1-\varphi_{v}\right)\cdot C^{c}:{\left[\left(1-\varphi_{v}\right)\cdot I+\varphi_{v}\cdot {\left(I-S\right)}^{-1}\right]}^{-1}$$

Since the composite domain is now a transversely isotropic material, the Eshelby tensor changes compared to the solution of an inhomogeneity in an isotropic matrix. A solution is given by Chiang for a cylinder inclusion with elliptic base in a transversely isotropic matrix, which is now represented with the composite domain [29]. The solution of Chiang is simplified and the components of the Eshelby tensor are explicitly given in Appendix A. The solution is identical to the solution of Withers in the case of a spheroidal inclusion ($a_{1}=a_{2}$, $a_{3}\neq a_{2}$) [30]. The unfolded Eshelby tensor for $a_{3}\overset{}{\to }\infty$ in Mandel (Kelvin) notation is given by:

(14) $$S=\left[\begin{array}{cccccc} S_{1111} & S_{1122} & S_{1133} & 0 & 0 & 0\\ S_{2211} & S_{2222} & S_{2233} & 0 & 0 & 0\\ 0 & 0 & 0 & 0 & 0 & 0\\ 0 & 0 & 0 & 2\;S_{2323} & 0 & 0\\ 0 & 0 & 0 & 0 & 2\;S_{3131} & 0\\ 0 & 0 & 0 & 0 & 0 & 2\;S_{1212} \end{array}\right]$$

With the elements $S_{3311}=S_{2233}=S_{1111}=0$ in the case of a cylinder with the axis aligned to the “3” direction. The fourth rank identity tensor I reduces to the 6×6 identity matrix for the Mandel (Kelvin) notation, with $\delta_{ij}=1$ for i=j:

(15) $$\begin{array}{cc} E_{ij}=\delta_{ij} & \mathrm{for}\;i,j=1...6 \end{array}$$

Equation (13) is now solved by replacing the fourth rank tensors with the tensors in Mandel (Kelvin) notation and application of matrix calculation rules:

(16) $$C^{mt}=\left(1-\varphi_{v}\right)\cdot C^{c}\cdot {\left[\left(1-\varphi_{v}\right)\cdot E+\varphi_{v}\cdot {\left(E-S\right)}^{-1}\right]}^{-1}$$

The inversion of Equation (16) (with application of ${\left(A\cdot B\right)}^{-1}=B^{-1}\cdot A^{-1})$ delivers the engineering constants of the composite material including voids:

(17) $${C^{mt}}^{-1}=\frac{1}{1-\varphi_{v}}\cdot \left[\left(1-\varphi_{v}\right)\cdot E+\varphi_{v}\cdot {\left(E-S\right)}^{-1}\right]\cdot {C^{c}}^{-1}$$

With superscript “*” denoting the effective properties of a composite containing voids after the two-step homogenization scheme and the subscript “11” indicating the element in row and line 1 of the inverse of the Mori–Tanaka stiffness matrix, the effective elastic constants of the void and composite domain can be calculated as follows, resulting in an orthotropic material:

(18) $$\begin{array}{ccc} E_{11}^{*}=1/{C_{11}^{mt}}^{-1} & E_{22}^{*}=1/{C_{22}^{mt}}^{-1} & E_{33}^{*}=1/{C_{33}^{mt}}^{-1} \end{array}$$

(19) $$\begin{array}{ccc} 2\;G_{23}^{*}=1/{C_{44}^{mt}}^{-1} & 2\;G_{13}^{*}=1/{C_{55}^{mt}}^{-1} & 2\;G_{12}^{*}=1/{C_{66}^{mt}}^{-1} \end{array}$$

(20) $$\begin{array}{ccc} \nu_{12}^{*}=-{C_{12}^{mt}}^{-1}/{C_{11}^{mt}}^{-1} & \nu_{13}^{*}=-{C_{13}^{mt}}^{-1}/{C_{11}^{mt}}^{-1} & \nu_{23}^{*}=-{C_{23}^{mt}}^{-1}/{C_{22}^{mt}}^{-1} \end{array}$$

The presented procedure is straightforward to implement to computer programs and allows for the estimation of the effective engineering constants including voids. However, for practical purposes, the explicit solutions of the engineering constants are favorable to analyze the dependence of the engineering constants on the shape of the void and of the void volume content. The explicit solutions are discussed in the next chapter.

## 3. Analytical Investigation

For the following analytical interpretation of the influence of the void shape and void content on the effective properties of a composite material including voids, the equations are solved explicitly. For the geometry of the voids, the aspect ratio *f* is defined as $f=a_{2}/a_{1}$.

### 3.1. Young’s Modulus in Fiber Direction

The Young’s modulus in fiber direction can be solved in a straightforward manner from Equation (17):

(21) $${C_{33}^{mt}}^{-1}=\frac{1}{1-\varphi_{v}}\cdot {C_{33}^{c}}^{-1}$$

That gives the final result for the fiber parallel modulus:

(22) $$E_{33}^{*}=E_{33}^{c}\cdot \left(1-\varphi_{v}\right)$$

Since the modulus of the composite domain is calculated with the adjusted fiber volume content, $E_{33}^{c}$ may be a function of the void content. As discussed in Section 2.1, $\varphi_{f}^{*}$ depends on the manufacturing process. Introducing the definition of the adjusted fiber volume content into Equation (22) and the mixing rule from Equation (2) results in:

(23) $$\begin{array}{c} E_{33}^{*}=\left\{\begin{array}{cc} E_{33,0}^{c}-E_{m}\cdot \varphi_{v} & e.g.,\;\text{for pultrusion or RTM processes}\\ E_{33,0}^{c}\cdot \left(1-\varphi_{v}\right) & e.g.,\;\text{for filament winding processes} \end{array}\right. \end{array}$$

With $E_{33,0}^{c}$ as the initial modulus calculated with the global fiber volume content. Since the Young’s modulus of the matrix is orders of magnitude smaller compared to the fiber or composite modulus, it is expected that the decrease of the Young’s modulus for RTM or pultrusion processes is smaller compared to filament winding processes. In this case the formula coincides with the mixing rule with a reduced matrix volume content in the composite domain. As expected for both processes, the effective Young’s modulus of a composite in fiber direction is linear dependent on the void volume content, but not of the shape of the void.

### 3.2. Shear Moduli

For the shear modulus in the plane “2−3” Equation (17) simplifies to:

(24) $${C_{44}^{mt}}^{-1}=\frac{1-2\;S_{2323}\cdot \left(1-\varphi_{v}\right)}{\left(1-2\;S_{2323}\right)\cdot \left(1-\varphi_{v}\right)}\cdot {C_{44}^{c}}^{-1}$$

The Eshelby tensor components are replaced by the components of Appendix A giving the final equation for $G_{23}^{*}$

(25) $$G_{23}^{*}=G_{23}^{c}\cdot \frac{f\cdot (1-\varphi_{v})}{f+\varphi_{v}}$$

The shear modulus in the “1−3” plane is derived analogous:

(26) $$G_{13}^{*}=G_{13}^{c}\cdot \frac{\frac{1}{f}\cdot \left(1-\varphi_{v}\right)}{\frac{1}{f}+\varphi_{v}}$$

Both moduli depend on the void volume content and the aspect ratio of the void. As expected, the shear modulus $G_{13}^{*}$ can be calculated with the same equation as $G_{23}^{*}$ except for the inverse of the aspect ratio *f*. For manufacturing processes in which the adjusted fiber volume content is changed by the void content, the shear moduli from the first homogenization step $G_{23}^{c}$ and $G_{13}^{c}$ are still functions of the void content. For the shear modulus $G_{13}^{c}$ this leads to the phenomenon that for low aspect ratios f<<1 a stiffening effect is predicted because of the higher fiber volume content in the composite domain. To emphasize this effect, Equation (26) is normalized with the initial modulus without any void and separated into the parts from the first and the second homogenization step:

(27) $$\frac{G_{13}^{*}}{G_{13,0}^{c}}=\underset{\begin{array}{c} \left(I\right)\;\mathrm{From}\;1\mathrm{st}\;\mathrm{step} \end{array}}{\underset{︸}{\frac{G_{13}^{c}\left(\varphi_{f}^{*}\right)}{G_{13,0}^{c}}}}\cdot \underset{\begin{array}{c} \left(\mathrm{II}\right)\;\mathrm{From}\;2\mathrm{nd}\;\mathrm{step} \end{array}}{\underset{︸}{\frac{\frac{1}{f}\cdot \left(1-\varphi_{v}\right)}{\frac{1}{f}+\varphi_{v}}}}$$

A stiffening effect can be observed, if the normalized properties are higher than 1, or equally, if the function

(28) $$F=\left(II\right)-\frac{1}{\left(I\right)}$$

reaches values higher than zero.

The same development is done for the shear modulus in the “1−2” plane, resulting in:

(29) $$G_{12}^{*}=G_{12}^{c}\cdot \frac{\left(1-2\;S_{1212}\right)\cdot \left(1-\varphi_{v}\right)}{1-2\;S_{1212}\cdot \left(1-\varphi_{v}\right)}$$

With replacing the component of the Eshelby tensor with Equation (A18) (Appendix A) and the components of the composite domain with the engineering constants (Equation (12)), Equation (29) can be reordered to:

(30) $$G_{12}^{*}=G_{12}^{c}\cdot \frac{\left(1+\nu_{12}^{c}\right)\cdot f\cdot \left(1-\varphi_{v}\right)}{\left(1-\nu_{23}^{c}\;\nu_{32}^{c}\right)\cdot {\left(1+f\right)}^{2}\cdot \varphi_{v}+\left(1+\nu_{12}^{c}\right)\cdot f\cdot \left(1-\varphi_{v}\right)};$$

For manufacturing processes with increasing fiber volume content in the composite domain, also the modulus $G_{12}^{*}$ may show a stiffening effect from the higher adjusted fiber volume content. The condition for the stiffening effect can be derived from Equation (28) by changing the terms (I) and (II) from the terms of Equation (30).

#### Approximate Solution for Out of Plane Shear Modulus

With $\nu_{23}^{c}\cdot \nu_{32}^{c}\ll 1$ Equation (30) simplifies to:

(31) $$G_{12}^{*}\approx G_{12}^{c}\cdot \frac{\left(1+\nu_{12}^{c}\right)\cdot f\cdot \left(1-\varphi_{v}\right)}{{\left(1+f\right)}^{2}\cdot \varphi_{v}+\left(1+\nu_{12}^{c}\right)\cdot f\cdot \left(1-\varphi_{v}\right)};$$

### 3.3. Young’s Modulus Transverse to Fiber Direction

Solving Equation (17) for the first entry delivers:

(32) $$\begin{array}{cc} {C_{11}^{mt}}^{-1}= & \frac{1}{1-\varphi_{v}}\cdot [\left(1-\varphi_{v}+\frac{\varphi_{v}}{\Delta }\cdot \left(S_{2222}-1\right)\right)\cdot {C_{11}^{c}}^{-1}\\ \; & -\frac{\varphi_{v}}{\Delta }\cdot S_{1122}\cdot {C_{21}^{c}}^{-1}\\ \; & -\frac{\varphi_{v}}{\Delta }\cdot \left(S_{1133}+S_{1122}\cdot S_{2233}-S_{1133}\cdot S_{2222}\right)\cdot {C_{31}^{c}}^{-1}] \end{array}$$

With Δ:

(33) $$\Delta =S_{1111}+S_{2222}-S_{1111}\cdot S_{2222}+S_{1122}\cdot S_{2211}-1$$

Finally, this leads to the result of the perpendicular Young’s modulus of the composite and void domain in the lamina plane:

(34) $$E_{11}^{*}=E_{11}^{c}\cdot \frac{\Delta \cdot (1-\varphi_{v})}{\Delta \cdot \left(1-\varphi_{v}\right)+\varphi_{v}\cdot \left(S_{2222}-1\right)+\varphi_{v}\cdot S_{1122}\cdot \nu_{12}^{c}+\varphi_{v}\cdot \Sigma_{11}\cdot \nu_{13}^{c}}$$

With:

(35) $$\Sigma_{11}=S_{1133}+S_{1122}\cdot S_{2233}-S_{1133}\cdot S_{2222}$$

The same development is done for the modulus in thickness direction “2”:

(36) $$E_{22}^{*}=E_{11}^{c}\cdot \frac{\Delta \cdot (1-\varphi_{v})}{\Delta \cdot \left(1-\varphi_{v}\right)+\varphi_{v}\cdot \left(S_{1111}-1\right)+\varphi_{v}\cdot S_{2211}\cdot \nu_{12}^{c}+\varphi_{v}\cdot \Sigma_{22}\cdot \nu_{23}^{c}}$$

With:

(37) $$\Sigma_{22}=S_{2233}+S_{1133}\cdot S_{2211}-S_{2233}\cdot S_{1111}$$

Giving the final results for both moduli:

(38) $$\begin{array}{ccc} E_{11}^{*} & = & E_{11}^{c}\cdot \frac{1-\varphi_{v}}{1+2\cdot f\cdot \varphi_{v}\cdot \left(1-E_{33}^{c}/E_{11}^{c}\cdot {\nu_{13}^{c}}^{2}\right)} \end{array}$$

(39) $$\begin{array}{ccc} E_{22}^{*} & = & E_{11}^{c}\cdot \frac{1-\varphi_{v}}{1+2\cdot \frac{1}{f}\cdot \varphi_{v}\cdot \left(1-E_{33}^{c}/E_{11}^{c}\cdot {\nu_{13}^{c}}^{2}\right)} \end{array}$$

Again, both moduli depend on the aspect ratio and of the void content.

#### Approximate Solution for Transverse Young’s Moduli

The Eshelby tensor components require $\frac{C_{12}^{c}}{C_{11}^{c}}$ and $\frac{C_{13}^{c}}{C_{11}^{c}}$:

(40) $$\begin{array}{ccc} \frac{C_{12}^{c}}{C_{11}^{c}} & = & \frac{\nu_{12}^{c}\cdot E_{11}^{c}+E_{33}^{c}\cdot \nu_{13}^{2}}{E_{11}^{c}-E_{33}^{c}\cdot {\nu_{13}^{c}}^{2}} \end{array}$$

(41) $$\begin{array}{ccc} \frac{C_{13}^{c}}{C_{11}^{c}} & = & \frac{E_{33}^{c}\cdot \nu_{13}^{c}\left(E_{11}^{c}\cdot \left({\nu_{12}^{c}}^{2}-1\right)+2\;E_{33}^{c}\cdot {\nu_{13}^{c}}^{2}\cdot \left(1+\nu_{12}^{c}\right)\right)}{\left(E_{11}^{c}-E_{33}^{c}\cdot {\nu_{13}^{c}}^{2}\right)\cdot \left(2\;E_{33}^{c}\cdot {\nu_{13}^{c}}^{2}+E_{11}^{c}\cdot \left(\nu_{12}^{c}-1\right)\right)} \end{array}$$

With ${\nu_{13}^{c}}^{2}\ll \frac{E_{11}^{c}}{E_{33}^{c}}$ they simplify to:

(42) $$\begin{array}{ccc} \frac{C_{12}^{c}}{C_{11}^{c}} & = & \nu_{12}^{c} \end{array}$$

(43) $$\begin{array}{ccc} \frac{C_{13}^{c}}{C_{11}^{c}} & = & \frac{E_{33}^{c}}{E_{11}^{c}}\cdot \left(\nu_{13}^{c}\cdot \left(1+\nu_{12}^{c}\right)\right) \end{array}$$

The ratios are introduced into Equations for the Eshelby tensor in the Appendix A and then into Equation (33). Since $\Sigma_{11}$ and $\Sigma_{22}$ are linear functions of the minor Poisson ratio $\nu_{13}^{c}$, the terms in Equations (34) and (36) for $\nu_{13}^{c}\;\Sigma_{ii}\;\varphi_{v}$ are omitted. After some further reordering the Young’s moduli can be easily calculated as:

(44) $$\begin{array}{ccc} E_{11}^{*} & = & E_{11}^{c}\cdot \frac{1}{f}\cdot \frac{1-\varphi_{v}}{\frac{1}{f}+2\;\varphi_{v}} \end{array}$$

(45) $$\begin{array}{ccc} E_{22}^{*} & = & E_{11}^{c}\cdot f\cdot \frac{1-\varphi_{v}}{f+2\;\varphi_{v}} \end{array}$$

As already seen for the shear moduli $G_{23}^{*}$ and $G_{13}^{*}$, the moduli can be determined by the inverse of the aspect ratio from each other.

### 3.4. Poisson Ratios

From Equation (18) the minor Poisson numbers are obtained. With the symmetry of the orthotropic stiffness tensor in Mandel (Kelvin) notation the other Poisson numbers are calculated. After reordering this gives the final result:

(46) $$\begin{array}{ccc} \nu_{12}^{*} & = & \frac{E_{11}^{c}\cdot \nu_{12}^{c}+E_{11}^{c}\cdot \varphi_{v}-E_{11}^{c}\cdot \nu_{12}^{c}\cdot \varphi_{v}-2\;E_{33}^{c}\cdot \varphi_{v}\cdot {\nu_{13}^{c}}^{2}}{E_{11}^{c}+2\;E_{11}^{c}\cdot f\cdot \varphi_{v}-2\;E_{33}^{c}\cdot f\cdot \varphi_{v}\cdot {\nu_{13}^{c}}^{2}} \end{array}$$

(47) $$\begin{array}{ccc} \nu_{21}^{*} & = & \frac{E_{11}^{c}\cdot \nu_{12}^{c}+E_{11}^{c}\cdot \varphi_{v}-E_{11}^{c}\cdot \nu_{12}^{c}\cdot \varphi_{v}-2\;E_{33}^{c}\cdot \varphi_{v}\cdot {\nu_{13}^{c}}^{2}}{E_{11}^{c}+2\;E_{11}^{c}\cdot 1/f\cdot \varphi_{v}-2\;E_{33}^{c}\cdot 1/f\cdot \varphi_{v}\cdot {\nu_{13}^{c}}^{2}} \end{array}$$

(48) $$\begin{array}{ccc} \nu_{31}^{*} & = & \nu_{32}^{*}=\nu_{31}^{c} \end{array}$$

(49) $$\begin{array}{ccc} \nu_{13}^{*} & = & \frac{E_{11}^{c}\cdot \nu_{13}^{c}}{E_{11}^{c}+2\;E_{11}^{c}\cdot f\cdot \varphi_{v}-2\;E_{33}^{c}\cdot f\cdot \varphi_{v}\cdot {\nu_{13}^{c}}^{2}} \end{array}$$

(50) $$\begin{array}{ccc} \nu_{23}^{*} & = & \frac{E_{11}^{c}\cdot \nu_{13}^{c}}{E_{11}^{c}+2\;E_{11}^{c}\cdot 1/f\cdot \varphi_{v}-2\;E_{33}^{c}\cdot 1/f\cdot \varphi_{v}\cdot {\nu_{13}^{c}}^{2}} \end{array}$$

The effective Poisson number $\nu_{31}^{*}$ and $\nu_{32}^{*}$ only depend on the result of the first homogenization step and remain unaffected by the shape of the void. In the case of a changed fiber volume content of the composite domain where Equation (1) is applied, the void content also changes the Poisson number. This result is identical to the result published for a void in an isotropic continuum [32]. $\nu_{12}^{*}$ and $\nu_{21}^{*}$ as well as $\nu_{13}^{*}$ and $\nu_{23}^{*}$ can be calculated from each other by use of the inverse of the aspect ratio *f* as shown for the Young’s moduli $E_{22}^{*}$ and $E_{11}^{*}$.

### 3.5. Limiting Cases

#### 3.5.1. Circular Basis

With the analytical expressions of Equations (22)–(37), it is now possible to estimate the resulting composite properties for the limiting cases of a cylindrical void shape with the same semi-axes ($a_{2}=a_{1}$). For the aspect ratio of f=1 the Eshelby tensor components reported by Chiang et al. are obtained [29]. The engineering constants of the composite with voids now result in:

(51) $$\begin{array}{ccc} E_{33}^{*} & = & E_{33}^{c}\cdot \left(1-\varphi_{v}\right) \end{array}$$

(52) $$\begin{array}{ccc} E_{11}^{*} & = & E_{11}^{c}\cdot \frac{1-\varphi_{v}}{1+2\cdot \varphi_{v}\cdot \left(1-E_{33}^{c}/E_{11}^{c}\cdot {\nu_{13}^{c}}^{2}\right)} \end{array}$$

(53) $$\begin{array}{ccc} E_{22}^{*} & = & E_{11}^{*} \end{array}$$

(54) $$\begin{array}{ccc} G_{13}^{*} & = & G_{13}^{c}\cdot \frac{1-\varphi_{v}}{1+\varphi_{v}} \end{array}$$

(55) $$\begin{array}{ccc} G_{23}^{*} & = & G_{13}^{*} \end{array}$$

(56) $$\begin{array}{ccc} G_{12}^{*} & = & G_{12}^{c}\cdot \frac{\left(1+\nu_{12}^{c}\right)\cdot \left(1-\varphi_{v}\right)}{4\left(1-\nu_{23}^{c}\;\nu_{32}^{c}\right)\cdot \varphi_{v}+\left(1+\nu_{12}^{c}\right)\cdot \left(1-\varphi_{v}\right)} \end{array}$$

For the void content of $\varphi_{v}=0$, the results for the engineering constants of the composite domain without any void are recovered.

#### 3.5.2. Inter Fiber Failure

With $a_{1}$ being much larger than $a_{2}$, the void acts like a crack and the state of an inter fiber failure perpendicular to the “2” direction is reached. It is expected that the moduli remain unaffected or are zero. For $f\overset{}{\to }0$ the Eshelby tensor components result in:

(57) $$\begin{array}{ccc} S_{1111}=S_{1122}=S_{1133}=S_{3131}=0 & S_{2211}=\frac{C_{12}^{c}}{C_{11}^{c}} & S_{2222}=1 \end{array}$$

(58) $$\begin{array}{ccc} S_{2233}=\frac{C_{13}^{c}}{C_{11}^{c}} & S_{2323}=\frac{1}{2} & S_{1212}=\frac{1}{2} \end{array}$$

Replacing the Eshelby tensor components into Equations (23), (25), (26), (30), (34) and (36) now gives the engineering constants:

(59) $$\begin{array}{ccc} E_{11}^{*}=E_{11}^{c} & E_{22}^{*}=0 & E_{33}^{*}=E_{33}^{c}\cdot \left(1-\varphi_{v}\right) \end{array}$$

(60) $$\begin{array}{ccc} G_{23}^{*}=0 & G_{13}^{*}=G_{13}^{c} & G_{12}^{*}=0 \end{array}$$

Since the void acts like a complete crack in the lamina plane “1−3”, only the moduli $G_{13}^{*}$ and $E_{11}^{*}$ remain the same as received from the first homogenization step. However, the definition of a void content for an inter fiber failure may seem questionable, although the analytic expressions above are still valid for the use of a void volume content of zero.

## 4. Numerical Examples

The method is applied to a material of glass fibers and epoxy resin with the properties of Table 1 taken from Huang and Zhou [37]. The glass fiber is assumed as isotropic.

**Figure 2:**
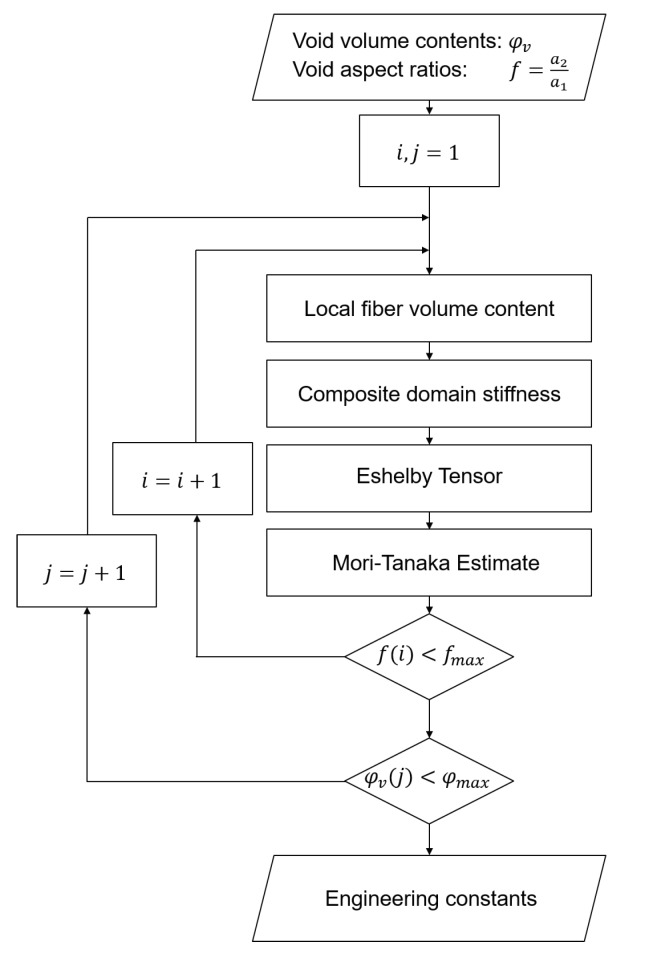
Flow chart of the implemented algorithm.

The method evaluates the resulting engineering constants for different volume contents and aspect ratios of the cylindrical voids with elliptic base for a global fiber volume content of 50%. The process is assumed to be an RTM process, in which the adjusted fiber volume content changes. The void volume content was varied in ten steps in the interval of 0% to 7% in the first homogenization step to calculate with Equations (1)–(9) the resulting properties of the composite domain. Then Equation (17) was applied to calculate the engineering constants of the composite and the void domain. The aspect ratio was varied in ten steps in the interval $\left[0.4,\;1.0\right]$. Both homogenization steps were implemented to the software Matlab, TheMathworks, USA. The flow chart of the implemented scheme is shown in Figure 2.

The numerical values for the first and the second homogenization step are listed exemplary in Table 2, Table 3 and Table 4 for one aspect ratio and one void volume content. The components of the stiffness matrix are given in Mandel (Kelvin) notation, which is different in the shear properties by a factor of 2 compared to the common Voigt notation.

**Figure 3:**
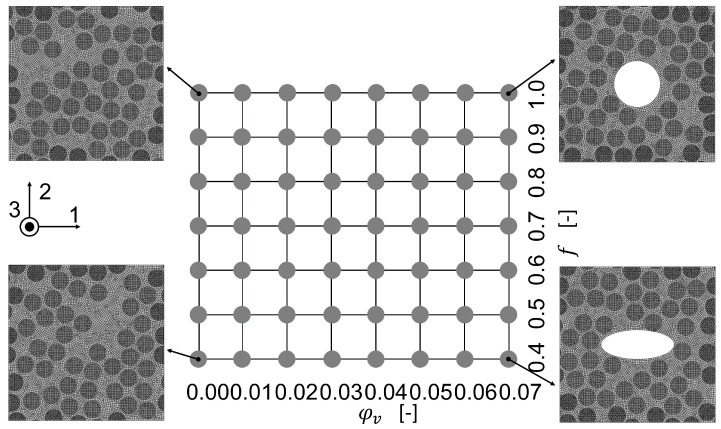
Finite Element experimental plan with four RVE microstructures.

The results of the method were compared to a representative volume element (RVE) in the finite element method which was solved with the implicit solver in Abaqus 2018, Simulia, USA. The algorithm for creation of the positions of the fibers was based on the Random sequential expansion algorithm proposed by Yang et al. [38], with one centered void. The void volume content was varied between 0% to 7% and the void aspect ratio was varied in the interval $\left[0.4,\;1.0\right]$. For the void volume content of 0% seven different fiber distributions were modelled to evaluate the quality of the homogenization in the first step. The plan of the numerical experiments is shown in Figure 3 including four RVE microstructures.

**Figure 4:**
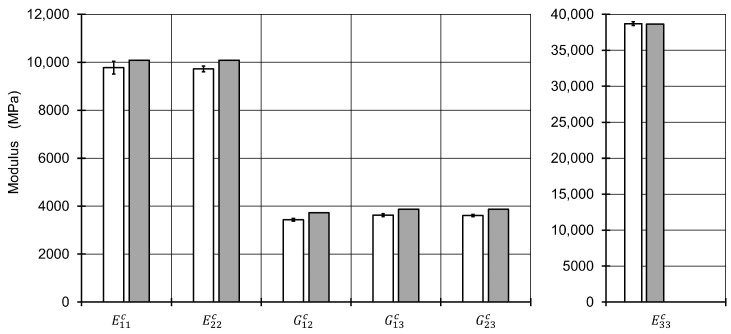
Resulting moduli of the first homogenization step calculated via the RVE microstructures (white bars) and via the used mixing rules (gray bars).

**Figure 5:**
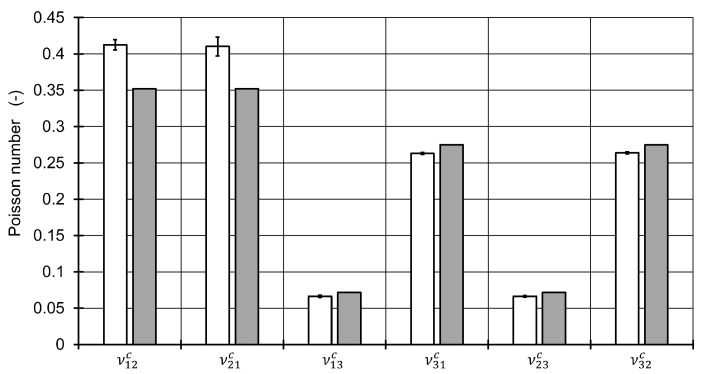
Resulting Poisson numbers of the first homogenization step calculated via the RVE microstructures (white bars) and via the used mixing rules (gray bars).

The fiber volume content was set a-priori to 50% with respect to the polygon shape of the meshed fibers inside the RVE. A total number of 50 fibers was used based on the investigations of Trias et al. [39]. The dimension in 1 and 2 direction of the RVE are set equal and reach a length of 17.67 times the fiber radius. The mesh for each of the 56 different microstructures was established by 32 nodes along the fiber circumferences for fibers inside the RVE after a mesh-convergence study. For fibers crossing an edge of the RVE, the fibers were cut and repeated at the opposite side of the RVE. Their circumferences were discretized with the same distance between the nodes as for uncut fibers inside the RVE. Element types for the matrix and the fibers inside the RVE were selected as linear hexagonal elements with reduced integration (Abaqus code C3D8R). Fibers crossing an edge were meshed with wedge elements (C3D6). This results in a total number of around 10,000 elements with 19,000 nodes with minor differences between each microstructure. Reference points were coupled via constraint equations to one side of the RVE in each direction to apply loads to the RVE. Opposing sides of the RVE were coupled by the periodic boundary conditions. The details and determination of stress/strain response of an RVE may be found elsewhere [40,41]. Material models for fiber and were assigned as linear elastic with the values of Table 1. For each of the microstructures, six load cases to obtain moduli and Poisson numbers were defined. With using 8 cores of an Intel Xeon CPU E5-2660 v3 at 2.6 GHz and 192 GB RAM, this leads to average calculation times of 19 seconds for each load step, resulting in a total calculation time of 6,400 seconds. In contrast, the method implemented to Matlab and solved with one core on an Intel Core i7-4710MQ CPU at 2.5 GHz and 16 GB RAM delivers all of the engineering constants in 0.01 seconds on a finer grid compared to the plan in Figure 3. The engineering constants of the seven microstructures without any void were compared with the ones from the mixing rules of the first homogenization step (Figure 4 and Figure 5). The highest relative difference is found for the shear modulus $G_{12}^{c}$, which is caused by the application of the mixing rule according to Christensen in the first homogenization step for the Poisson number $\nu_{12}^{c}$[18]. All other moduli show a reasonable agreement. However, different standard deviations between the moduli $G_{23}^{c}$ and $G_{13}^{c}$ as well as for $E_{11}^{c}$, $E_{22}^{c}$ and the Poisson numbers $\nu_{12}^{c}$ and $\nu_{21}^{c}$ show that a higher number of different RVE model calculations is advisable for the determination of engineering constants via a finite element model.

**Figure 6:**
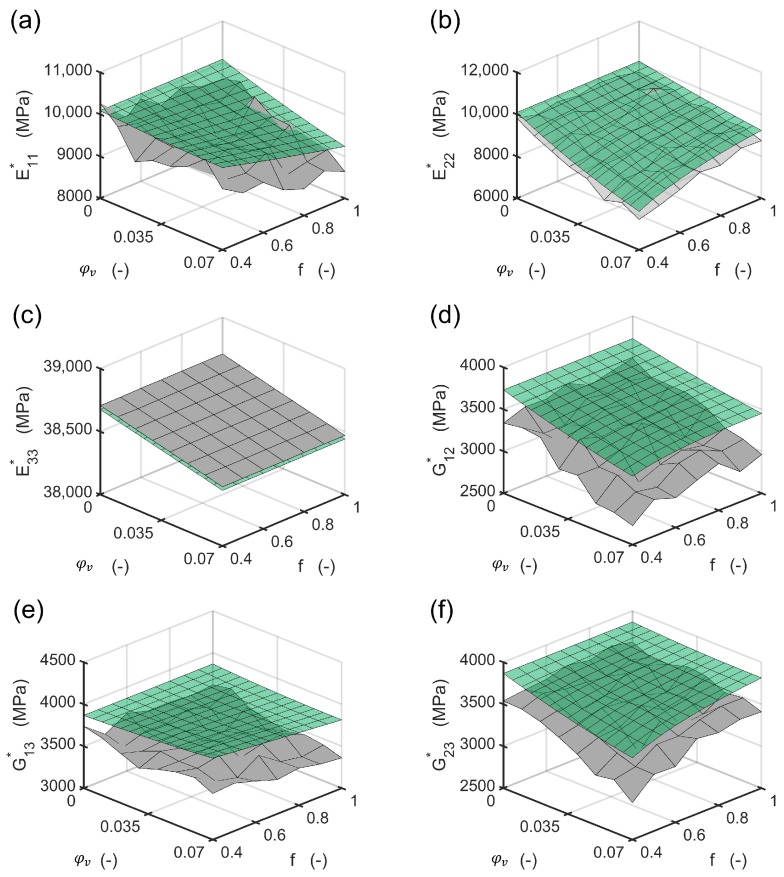
Resulting moduli calculated with the FEM RVE (gray surface) compared to the moduli calculated with the method presented (green surface): (**a**) transverse Young’s modulus “1” direction; (**b**) transverse Young’s modulus “2” direction; (**c**) fiber parallel Young’s modulus “3” direction; (**d**) transverse shear modulus; (**e**) in plane shear modulus; (**f**) out of plane shear modulus.

**Figure 7:**
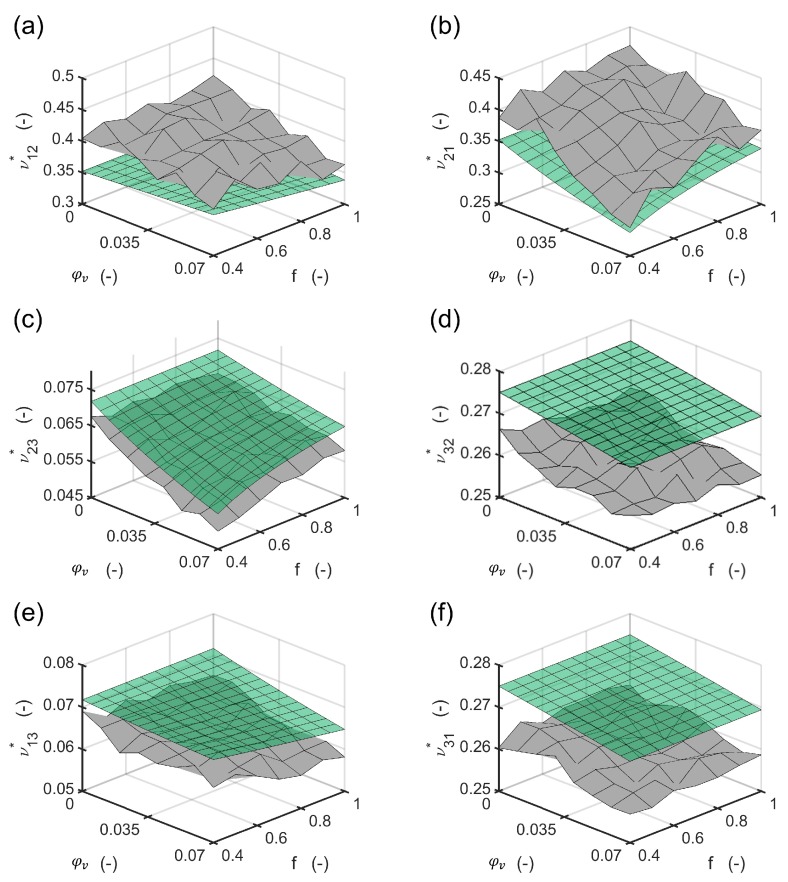
Resulting Poisson numbers calculated with the FEM RVE (gray surface) compared to the Poisson numbers calculated with the method presented (green surface): (**a**) transverse Poisson number for tension in “1” and reaction in “2” direction; (**b**) transverse Poisson number for tension in “2” and reaction in “1” direction; (**c**) minor out of plane Poisson number; (**d**) major out of plane Poisson number; (**e**) minor in plane Poisson number; (**f**) major in plane Poisson number.

For all engineering constants, the slope of the change in the properties shows a reasonable agreement between the method presented and the finite element solution (Figure 6 and Figure 7). Numerical improvements for the method presented can be achieved using different mixing rules in the first homogenization step.

### Approximate Calculation of Moduli

**Figure 8:**
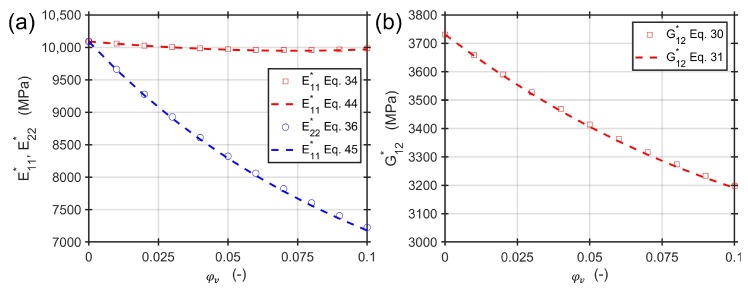
Comparison of approximate solutions and the exact solutions depending on void volume content and aspect ratio of 0.4: (**a**) Young’s moduli $E_{11}^{*}$ and $E_{22}^{*}$; (**b**) shear modulus $G_{12}^{*}$.

The approximate Equations (31), (44), and (45) for an aspect ratio of f=0.4 and a fiber volume content of 50% in the range of 0% and 10% of void volume content are compared to the exact solutions of Equations (30), (34), and (36). The materials are the same as in the previous section. Figure 8 shows an excellent agreement of both calculation approaches under the assumptions made. The approximate solutions allow for a simple and straightforward way to estimate the engineering constants for a composite material containing voids of different aspect ratios and volume contents.

## 5. Comparison with Experimental Data from Literature

The method presented is compared to experimental data from literature. The investigation of Olivier et al. [42] is based on pre-preg material from two different types, namely T2H 132 300 EH 25 (A) and R922 1C 12K (B). Both materials are unidirectional carbon fiber epoxy materials with initial fiber volume contents of $\varphi_{f}=60\%$ before manufacturing of specimens. The void contents’ range is $\varphi_{v}=0.3\%$ to 10.3%. For material “A” they report the void positions of larger voids between the plies. Together with the measured dimensions of the voids, they can be modelled as meso-voids. The aspect ratios of the ellipsoid/cylindrical voids range from for f=1 for lower void contents up to f=0.3 for higher void contents. Since the global fiber volume content for all specimens is reported to be approximately 63% and no significant influence of the void content on the Young’s modulus in fiber direction was observed, the fiber volume content of the composite domain is adjusted with the formula (1).

In the calculation, the input parameters from Soden et al. [43] in Table 5 for the fiber were used. For the EH 25 epoxy resin system the datasheet value for the Young’s modulus of E=3700 MPa [44] was used. Poisson number was assumed as ν=0.35.

**Figure 9:**
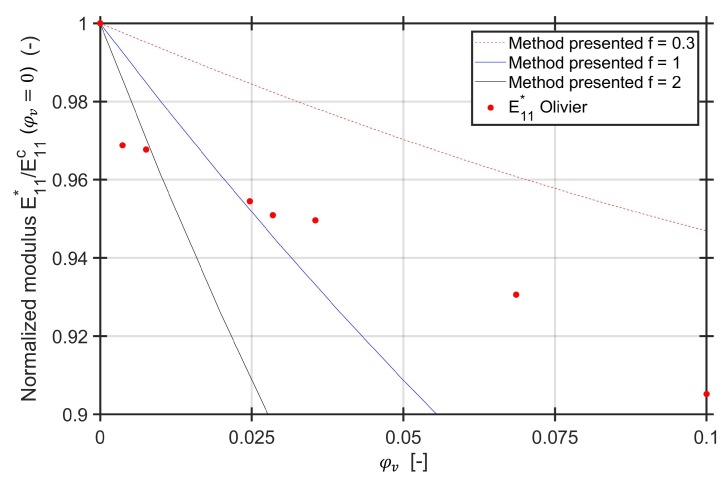
Normalized Young’s modulus $E_{11}^{*}$ as reported by Olivier et al. [42] and calculated with the method presented.

All values are normalized to the first numerical value for $\varphi_{v}=0$. The plot of the normalized values compared to the experimental values is displayed in Figure 9 for three different aspect ratios ranging from f=0.3 up to f=2 including f=1 for the cylindrical void with circular basis.

The method presented for the aspect ratio of f=0.3 follows the slope of decreasing modulus with increasing void content for specimens with voids. However, the loss in stiffness for moderate void volume contents compared to a void free specimen cannot be calculated with the aspect ratio of f=0.3. Since for lower void contents the authors describe spherical voids, which are modelled within the scope of this method as cylindrical with circular basis, the Young’s modulus is also calculated for the aspect ratio of f=1. This aspect ratio coincides with the experimental results of the intermediate void contents. For reaching the steep loss in stiffness, an aspect ratio of greater than f=2 is required. According to the described results, this aspect ratio does not seem realistic. However, also for the longitudinal modulus (which is not shown in this paper), the experimental results showed some scatter. Normalization of the scattered values to the first value of a void free specimen may lead to a misinterpretation of the slope of the experimental results. Furthermore, the fiber volume content is only reported once, which leads to an uncertainty if the void free specimens have the same global fiber volume content as the specimens with voids.

Harper et al. investigated the influence of voids on the mechanical properties of AS4/3502 carbon fiber-reinforced epoxy pre-preg [45]. They used unidirectional specimens with void contents ranging from 0.2% and 6.0% to measure Young’s moduli in fiber parallel and fiber perpendicular direction as well as the in-plane shear modulus and major in-plane Poisson number. However, results are only plotted for void contents higher than 1%. Since different void contents were achieved by different pressure profiles in an autoclave process and no values are reported, the formula for an unchanged adjusted fiber volume content is applied. The shape of the void was also not investigated. Therefore, the voids are assumed as cylindrical with circular basis (f=1). Since the modulus $E_{11}^{*}$ is reported as absolute value, the first micromechanical homogenization step is omitted and a void free modulus of $E_{11}^{c}=$ 11,400 MPa is assumed. Material parameters for the AS4 fibers differ significantly through literature [43,46,47]. The results of the shear modulus clearly disagree with other results reported [43,48]. Therefore, only the modulus $E_{11}^{*}$ is compared to the results of the method presented.

**Figure 10:**
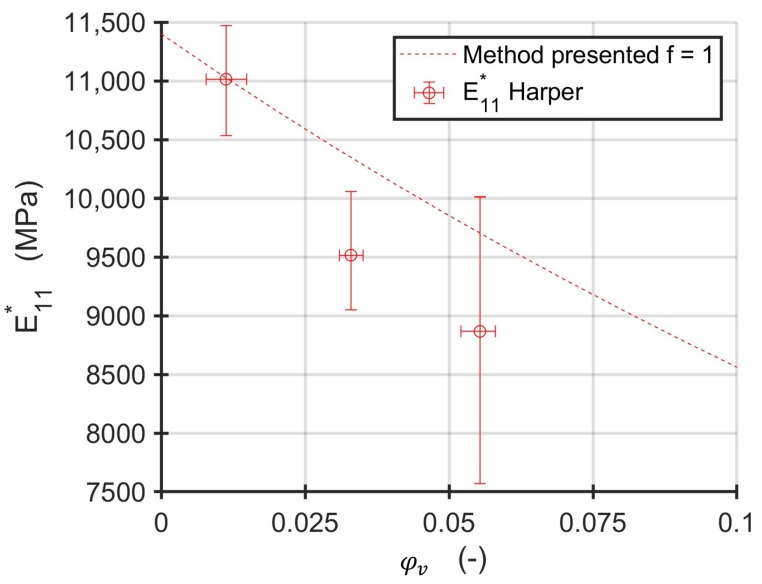
Young’s modulus $E_{11}^{*}$ as reported by Harper et al. [45] and calculated with the method presented.

The calculated fiber transverse modulus as shown in Figure 10 is in good agreement with the scatter band of the results of Harper [45], although the information on the manufacturing process and of the voids’ morphology is not available. This also may contribute to the high scatter in the void containing specimens, if different conditions on the processing of the panels were present. Since the assumption for the adjusted fiber volume content give bounds for different processing routes, real manufacturing processes may show a behavior in between. The analytically shown stiffening effect for high void contents and low aspect ratios also increases the difficulty of the interpretation of experimental results. Therefore, void morphology and manufacturing boundary conditions should be taken into consideration for a stiffness estimation of composites with voids.

## 6. Conclusions

The method presented allows for the calculation of the moduli of a composite material containing elliptic cylindrical voids. Due to the explicit formulation of all moduli and Poisson numbers, the calculation is straightforward, and orders of magnitude faster compared to a finite element solution. The method is not limited to low fiber volume contents, since a semi-empirical rule of mixture is used in the first step and the Mori–Tanaka estimate in the second step for an inclusion in a transversely isotropic material. The derived solutions allow for a direct calculation of the engineering constants without the calculation of the Eshelby tensor. From the analytical investigation, it is concluded that the moduli depend on the aspect ratio of the void in fiber transverse direction and of the void content. With the introduction of the adjusted fiber volume content in the first step, a stiffening effect is predicted for the transverse moduli depending on the manufacturing process. A lower reduction with increasing void volume content of the fiber parallel modulus is expected e.g., for the RTM process compared to processes that have no control of thickness or global fiber volume content. No influence of a void shape can be seen for the major Poisson number in the lamina and the thickness plane.

In experimental data from literature, void morphology and the manufacturing process are frequently not considered to be parameters. Since it could be shown that the voids’ morphology has an influence on the resulting engineering constants it should be considered to be additional quality parameter for fiber-reinforced composites. The manufacturing process also influences the resulting engineering constants and should be considered. The comparison of the method presented to experimental data from literature shows a reasonable agreement with the slope of the modulus decrease. However, differences may occur from the unknown distribution of the void morphology in the experiments and the details of the manufacturing process. In laboratory testing this could also contribute to the high scatter of experimental data. In future works the method can be expanded to incorporate different distributions of aspect ratios of the voids to obtain a prediction closer to experimental values. Also, different mixing rules or experimental data from void free specimens can be used in the first homogenization step to improve the prediction of engineering constants.

## Figures and Tables

**Table 1:** Input material properties.

	Young’s Modulus	Poisson Ratio	Global Volume Content
Epoxy resin	3350 MPa	0.35	50%
Glass fiber	74,000 MPa	0.2	50%

**Table 2:** First homogenization step for $\varphi_{v}=7\%$ and $\varphi_{f}=50\%$.

Variable	Value	Variable	Value
$\varphi_{f}$	50%	$\varphi_{v}$	7%
$\varphi_{f}^{*}$	53.76%		
$E_{11}^{c}=E_{22}^{c}$	11,295 MPa	$E_{33}^{c}$	41,334 MPa
$G_{13}^{c}=G_{23}^{c}$	4388 MPa	$G_{12}^{c}$	4210 MPa
$\nu_{12}^{c}$	0.342	$\nu_{13}^{c}$	0.0736
$\nu_{31}^{c}$=$\nu_{32}^{c}$	0.269		
$C_{11}^{c}$	13,336 MPa	$C_{33}^{c}$	43,982 MPa
$C_{12}^{c}$	4916 MPa	$C_{13}^{c}$	4916 MPa
$C_{44}^{c}$	8777 MPa	$C_{66}^{c}$	8419 MPa

**Table 3:** Second homogenization step for an aspect ratio of f=0.4.

Variable	Value	Variable	Value
$S_{1111}$	0.4254	$S_{2222}$	0.8539
$S_{1122}$	−0.0343	$S_{2211}$	0.1237
$S_{1133}$	0.1053	$S_{2233}$	0.2633
$S_{3131}$	0.1429	$S_{2323}$	0.3571
$S_{1212}$	0.3603		
$C_{11}^{mt}$	11,526 MPa	$C_{12}^{mt}$	3525 MPa
$C_{22}^{mt}$	9016 MPa	$C_{13}^{mt}$	4054 MPa
$C_{33}^{mt}$	40,442 MPa	$C_{23}^{mt}$	3378 MPa
$C_{44}^{mt}$	6947 MPa	$C_{55}^{mt}$	7940 MPa
$C_{66}^{mt}$	6632 MPa		

**Table 4:** Final results for the engineering constants after the second homogenization step.

Engineering Constant	Value
$E_{11}^{*}$	9958 MPa
$E_{22}^{*}$	7821 MPa
$E_{33}^{*}$	38,441 MPa
$G_{23}^{*}$	3473 MPa
$G_{13}^{*}$	3970 MPa
$G_{12}^{*}$	3316 MPa
$\nu_{12}^{*}$	0.365 MPa
$\nu_{21}^{*}$	0.287 MPa
$\nu_{23}^{*}$	0.0548 MPa
$\nu_{13}^{*}$	0.0698 MPa
$\nu_{31}^{*}$=$\nu_{32}^{*}$	0.269 MPa

**Table 5:** Input material properties of fiber for the comparison between Young’s modulus $E_{11}^{*}$ calculated by the method presented to the data presented by Olivier et al. [42].

Parameter	Value
Transverse Young’s modulus $E_{f,11}$	15,000 MPa
In-plane shear modulus $G_{f,23}$	15,000 MPa
Transverse shear modulus $G_{f,12}$	7000 MPa
Major Poisson ratio $\nu_{f,31}$	0.20
Minor Poisson ratio $\nu_{f,12}$	0.071

## Appendix A

The closed form solution of the Eshelby tensor is given following the solution according to Chiang [29]. Since the fiber orientation is in “3” direction, the solution for $a_{3}\overset{}{\to }\infty$ and $a_{2}\neq a_{1}$ is obtained for the nonzero components depending on the axis $a_{2}$ and $a_{1}$. The used input stiffness is from the first quadrant of the Mandel (Kelvin) notation of the stiffness tensor. The values in the first quadrant are identical to the ones of the more common Voigt notation:

(A1) $$\begin{array}{ccc} S_{1111} & = & \frac{a_{2}}{a_{1}+a_{2}}+\frac{a_{1}\cdot a_{2}}{{\left(a_{1}+a_{2}\right)}^{2}}\cdot \frac{C_{11}^{c}+C_{12}^{c}}{2\;C_{11}^{c}} \end{array}$$

(A2) $$\begin{array}{ccc} S_{1122} & = & \frac{a_{2}}{a_{1}+a_{2}}\cdot \frac{C_{12}^{c}}{C_{11}^{c}}-\frac{a_{1}\cdot a_{2}}{{\left(a_{1}+a_{2}\right)}^{2}}\cdot \frac{C_{11}^{c}+C_{12}^{c}}{2\;C_{11}^{c}} \end{array}$$

(A3) $$\begin{array}{ccc} S_{1133} & = & \frac{a_{2}}{a_{1}+a_{2}}\cdot \frac{C_{13}^{c}}{C_{11}^{c}} \end{array}$$

(A4) $$\begin{array}{ccc} S_{2211} & = & \frac{a_{1}}{a_{2}}\cdot \frac{a_{2}}{a_{1}+a_{2}}\cdot \frac{C_{12}^{c}}{C_{11}^{c}}-\frac{a_{1}\cdot a_{2}}{{\left(a_{1}+a_{2}\right)}^{2}}\cdot \frac{C_{11}^{c}+C_{12}^{c}}{2\;C_{11}^{c}} \end{array}$$

(A5) $$\begin{array}{ccc} S_{2222} & = & \frac{a_{1}}{a_{2}}\cdot \frac{a_{2}}{a_{1}+a_{2}}+\frac{a_{1}\cdot a_{2}}{{\left(a_{1}+a_{2}\right)}^{2}}\cdot \frac{C_{11}^{c}+C_{12}^{c}}{2\;C_{11}^{c}} \end{array}$$

(A6) $$\begin{array}{ccc} S_{2233} & = & \frac{a_{1}}{a_{2}}\cdot \frac{a_{2}}{a_{1}+a_{2}}\cdot \frac{C_{13}^{c}}{C_{11}^{c}} \end{array}$$

(A7) $$\begin{array}{ccc} S_{2323} & = & \frac{1}{2}\cdot \frac{a_{1}}{a_{1}+a_{2}} \end{array}$$

(A8) $$\begin{array}{ccc} S_{3131} & = & \frac{1}{2}\cdot \frac{a_{2}}{a_{1}+a_{2}} \end{array}$$

(A9) $$\begin{array}{ccc} S_{1212} & = & \frac{1}{2}-\frac{a_{1}\cdot a_{2}}{{\left(a_{1}+a_{2}\right)}^{2}}\cdot \frac{C_{11}^{c}+C_{12}^{c}}{2\;C_{11}^{c}} \end{array}$$

Or equivalently with the use of the aspect ratio $f=\frac{a_{2}}{a_{1}}$:

(A10) $$\begin{array}{ccc} S_{1111} & = & \frac{f}{1+f}+\frac{f}{{\left(1+f\right)}^{2}}\cdot \frac{C_{11}^{c}+C_{12}^{c}}{2\;C_{11}^{c}} \end{array}$$

(A11) $$\begin{array}{ccc} S_{1122} & = & \frac{f}{1+f}\cdot \frac{C_{12}^{c}}{C_{11}^{c}}-\frac{f}{{\left(1+f\right)}^{2}}\cdot \frac{C_{11}^{c}+C_{12}^{c}}{2\;C_{11}^{c}} \end{array}$$

(A12) $$\begin{array}{ccc} S_{1133} & = & \frac{f}{1+f}\cdot \frac{C_{13}^{c}}{C_{11}^{c}} \end{array}$$

(A13) $$\begin{array}{ccc} S_{2211} & = & \frac{1}{1+f}\cdot \frac{C_{12}^{c}}{C_{11}^{c}}-\frac{f}{{\left(1+f\right)}^{2}}\cdot \frac{C_{11}^{c}+C_{12}^{c}}{2\;C_{11}^{c}} \end{array}$$

(A14) $$\begin{array}{ccc} S_{2222} & = & \frac{1}{1+f}+\frac{f}{{\left(1+f\right)}^{2}}\cdot \frac{C_{11}^{c}+C_{12}^{c}}{2\;C_{11}^{c}} \end{array}$$

(A15) $$\begin{array}{ccc} S_{2233} & = & \frac{1}{1+f}\cdot \frac{C_{13}^{c}}{C_{11}^{c}} \end{array}$$

(A16) $$\begin{array}{ccc} S_{2323} & = & \frac{1}{2}\cdot \frac{1}{1+f} \end{array}$$

(A17) $$\begin{array}{ccc} S_{3131} & = & \frac{1}{2}\cdot \frac{f}{1+f} \end{array}$$

(A18) $$\begin{array}{ccc} S_{1212} & = & \frac{1}{2}-\frac{f}{{\left(1+f\right)}^{2}}\cdot \frac{C_{11}^{c}+C_{12}^{c}}{2\;C_{11}^{c}} \end{array}$$

## References

[1] Lenoe E. (1970). Effect of Voids on Mechanical Properties of Graphite Fiber Composites.

[2] Hagstrand P.O., Bonjour F., Månson J.A. (2005). The influence of void content on the structural flexural performance of unidirectional glass fibre reinforced polypropylene composites. Compos. Part A Appl. Sci. Manuf..

[3] Varna J., Joffe R., Berglund L.A., Lundström T.S. (1995). Effect of voids on failure mechanisms in RTM laminates. Compos. Sci. Technol..

[4] Gürdal Z., Tomasino A.P., Biggers S.B. (1991). Effects of processing induced defects on laminate response: Interlaminar tensile strength. SAMPE J..

[5] Liu H., Wen W., Su X., Engler-Pinto C., Kang H. (2017). Study on Fatigue Behaviors of Porous T300/924 Carbon Fiber Reinforced Polymer Unidirectional Laminates. SAE Int. J. Mater. Manuf..

[6] Mehdikhani M., Gorbatikh L., Verpoest I., Lomov S.V. (2018). Voids in fiber-reinforced polymer composites: A review on their formation, characteristics, and effects on mechanical performance. J. Compos. Mater..

[7] Peng X., Hu N., Zheng H., Fukunaga H. (2009). Evaluation of mechanical properties of particulate composites with a combined self-consistent and Mori–Tanaka approach. Mech. Mater..

[8] Kushch V.I., Sevostianov I. (2016). The “rigorous” Maxwell homogenization scheme in 2D elasticity: Effective stiffness tensor of composite with elliptic inhomogeneities. Mech. Mater..

[9] Barboura S., Ramtani S. (2009). Overall Mechanical Properties of Particulate Porous Composites Following Two-Step Homogenization Scheme. Appl. Mech. Mater..

[10] Cohen L., Ishai O. (1967). The Elastic Properties of Three-Phase Composites. J. Compos. Mater..

[11] Hori M., Nemat-Nasser S. (1994). Double-Inclusion Model and Overall Moduli of Multi-Phase Composites. J. Eng. Mater. Technol..

[12] Hill R. (1963). Elastic properties of reinforced solids: Some theoretical principles. J. Mech. Phys. Solids.

[13] Younes R., Hallal A., Fardoun F., Hajj F. (2012). Comparative Review Study on Elastic Properties Modeling for Unidirectional Composite Materials. Composites and Their Properties.

[14] Wall P. (1997). A Comparison of homogenization, Hashin-Shtrikman bounds and the Halpin-Tsai Equations. Appl. Math..

[15] McLaughlin R. (1977). A study of the differential scheme for composite materials. Int. J. Eng. Sci..

[16] Norris A.N. (1985). A differential Scheme for the effective moduli of Composites. Mech. Mater..

[17] Craig P.D., Summerscales J. (1988). Poisson’s Ratios in Glass Fibre Reinforced Plastics. Compos. Struct..

[18] Christensen R.M. (1998). The Numbers of Elastic Properties and Failure Parameters for Fiber Composites. J. Eng. Mater. Technol..

[19] Philippidis T.P., Theocaris P.S. (1994). The Transverse Poisson’s Ratio in Fiber Reinforced Laminae by Means of a Hybrid Experimental Approach. J. Compos. Mater..

[20] Tandon G.P., Weng G.J. (1984). The effect of aspect ratio of inclusions on the elastic properties of unidirectionally aligned composites. Polym. Compos..

[21] Mura T., Cheng P.C. (1977). The Elastic Field Outside an Ellipsoidal Inclusion. J. Appl. Mech..

[22] Benveniste Y. (1987). A new approach to the application of Mori-Tanaka’s theory in composite materials. Mech. Mater..

[23] Ju J.W., Chen T.M. (1994). Micromechanics and effective moduli of elastic composites containing randomly dispersed ellipsoidal inhomogeneities. Acta Mech..

[24] Tucker C.L., Liang E. (1999). Stiffness predictions for unidirectional short-fiber composites: Review and evaluation. Compos. Sci. Technol..

[25] Bleier A. (2011). Prüfverfahren zur Ermittlung exakter Werkstoffkennwerte einer Unidirektionalen Schicht unter Besonderer Berücksichtigung Physikalischer Nichtlinearitäten. Ph.D. Thesis.

[26] Huang H., Talreja R. (2005). Effects of void geometry on elastic properties of unidirectional fiber reinforced composites. Compos. Sci. Technol..

[27] Farouk A., Langrana N.A., Weng G.J. (1992). Modulus prediction of a cross-ply fiber reinforced fabric composite with voids. Polym. Compos..

[28] Chao L.P., Huang J.H. (1999). Prediction of Elastic Moduli of Porous Materials with Equivalent Inclusion Method. J. Reinf. Plast. Compos..

[29] Chiang C.R. (2017). On Eshelby’s tensor in transversely isotropic materials. Acta Mech..

[30] Withers P.J. (1989). The determination of the elastic field of an ellipsoidal inclusion in a transversely isotropic medium, and its relevance to composite materials. Philos. Mag. A.

[31] Nikishkov Y., Airoldi L., Makeev A. (2013). Measurement of voids in composites by X-ray Computed Tomography. Compos. Sci. Technol..

[32] Nemat-Nasser S., Hori M. (1993). Micromechanics: Overall Properties of Heterogeneous Materials.

[33] Christensen R.M. (1990). A critical Evaluation for a Class of Micro-Mechanics Models. J. Mech. Phys. Solids.

[34] Bennoura M., Aboutajeddine A. (2016). Double-Inclusion Model and Overall Moduli of Multi-Phase Composites. J. Reinf. Plast. Compos..

[35] Brannon R.M. (2018). Rotation, Reflection and Frame Changes: Orthogonal Tensors in Computational Engineering Mechanics.

[36] Dormieux L., Kondo D., Ulm F.J. (2006). Microporomechanics.

[37] Huang Z.M., Zhou Y.X. (2013). Correlation of the bridging model predictions for triaxial failure strengths of composites with experiments. J. Compos. Mater..

[38] Yang L., Yan Y., Ran Z., Liu Y. (2013). A new method for generating random fibre distributions for fibre reinforced composites. Compos. Sci. Technol..

[39] Trias D., Costa J., Turon A., Hurtado J. (2006). Determination of the critical size of a statistical representative volume element (SRVE) for carbon reinforced polymers. Acta Mater..

[40] Trias D., Costa J., Mayugo J.A., Hurtado J.E. (2006). Random models versus periodic models for fibre reinforced composites. Comput. Mater. Sci..

[41] Barello R.B., Lévesque M. (2008). Comparison between the relaxation spectra obtained from homogenization models and finite elements simulation for the same composite. Int. J. Solids Struct..

[42] Olivier P., Cottu J.P., Ferret B. (1995). Effects of cure cycle pressure and voids on some mechanical properties of carbon/epoxy laminates. Composites.

[43] Soden P.D., Hinton M.J., Kaddour A.S. (1998). Lamina properties, lay-up configurations and loading conditions for a range of fibre reinforced composite laminates. Compos. Sci. Technol..

[44] HexPly EH25 Hexcel Product Data Sheet. https://www.hexcel.com/user_area/content_media/raw/HexPly_EH25_eu_DataSheet.pdf.

[45] Harper B.D., Staab G.H., Chen R.S. (1987). A Note on the Effects of Voids Upon the Hygral and Mechanical Properties of AS4/3502 Graphite/Epoxy. J. Compos. Mater..

[46] Mishra K.D., El-Hajjar R.F. (2012). Non-linear strain invariant failure approach for fibre reinforced composite materials. Int. J. Mater. Struct. Integr..

[47] King T.R., Blackketter D.M., Walrath D.E., Adams D.F. (1992). Micromechanics Prediction of the Shear Strength of Carbon Fiber/Epoxy Matrix Composites: The Influence of the Matrix and Interface Strengths. J. Compos. Mater..

[48] Banks-Sills L. (2015). Interface fracture mechanics: Theory and experiment. Int. J. Fract..

